# Chest tube insertion strategy in uniportal thoracoscopic pulmonary resection: A propensity score-matched study

**DOI:** 10.1097/MD.0000000000046467

**Published:** 2025-12-19

**Authors:** Min Hu, Jing Li, Baochen Zhao, Haoran Sun

**Affiliations:** a Department Thoracic Surgery, The Second Affiliated Hospital of Wannan Medical College, Wuhu, Anhui, China; b Department of Medicine, Anhui Health College, Chizhou, Anhui China; c Economic and Technological Development Zone, Anhui Medical University, Graduate School, Hefei, Anhui, China.

**Keywords:** chest tube, postoperative pain, uniportal thoracoscopic, U-VATS

## Abstract

This study aimed to compare a modified chest tube drainage strategy with the traditional method in uniportal thoracoscopic pulmonary resection (U-VATS). We retrospectively analyzed 206 U-VATS patients treated at The Second Affiliated Hospital of Wannan Medical College (January 2022–December 2023). Patients were divided into a modified group (n = 46) and a traditional group (n = 160) based on drainage strategy. Propensity score matching (1:1) yielded 80 patients for comparison. Baseline characteristics (age, gender, smoking, BMI, lung function, resection extent, etc) showed no significant differences (*P* > .05). The modified group demonstrated superior outcomes: lower postoperative pain scores and Chronic postsurgical pain (*P* < .05), shorter extubation operation time, fewer extubation-related complications, reduced rescue analgesia needs, and better wound healing at the drainage site (*P* < .05). No differences were observed in operative time, drainage duration, volume, or hospital stay (*P* > .05). The modified drainage strategy is safe and equally effective for fluid management but reduces postoperative pain, accelerates extubation operation, and minimizes complications versus traditional methods. It may optimize recovery in U-VATS patients.

## 1. Introduction

In recent years, with the widespread adoption and refinement of thoracoscopic techniques, the number of incisions for video-assisted thoracoscopic surgery (VATS) has progressively decreased from the initial 3 to 4 to a single port, known as uniportal VATS.^[1]^ Compared to traditional multiport VATS lung resections, uniportal VATS results in less postoperative incision pain and reduced chest wall numbness; it provides a surgical view akin to open thoracotomy and offers more aesthetically pleasing wounds.^[2,3]^ However, to monitor potential complications such as bleeding, air leaks, and to prevent pleural effusion or pneumothorax while promoting lung re-expansion, a chest drainage tube is routinely placed postoperatively. The relatively rigid and large diameter of these tubes can introduce complications, including incision pain, exudate, poor wound healing, and infections, particularly impeding rapid postoperative recovery due to poor wound healing.^[4,5]^ Recent reports suggest modifications in the shape, diameter, and number of drainage tubes, as well as special skin suturing techniques and tube fixation methods, to minimize discomfort and enhance cosmetic wound healing. Nevertheless, there is no standardized method for effectively placing a chest drainage tube.^[4,6,7]^ During the suturing process, appropriate tension, skin temperature, tissue swelling, and adequate alignment of the skin at the incision site are crucial for wound healing. Thus, we have developed an improved method for the placement of drainage tubes, which not only achieves the drainage efficacy of traditional methods but also significantly reduces postoperative pain and enhances drainage tube site wound healing grade.

## 2. Materials and methods

A retrospective analysis was conducted on the clinical data of 206 patients treated with uniportal thoracoscopic surgery for bullous lung disease or pulmonary nodules between January 2022 and December 2023 at The Second Affiliated Hospital of Wannan Medical College. All procedures were successfully performed by the same surgical team. Patients were divided into 2 groups based on the method of chest drainage tube insertion: 46 patients underwent a modified insertion technique (experimental group), and 160 patients followed the traditional method (control group). All participants had signed informed consent prior to the study, which was ethically approved by The Second Affiliated Hospital of Wannan Medical College Ethics Committee (WYEFYS202104). All patients provided written informed consent before inclusion.

Inclusion criteria included: no contraindications for surgery identified during preoperative evaluation; elective surgery patients; successful completion of uniportal thoracoscopic surgery, performed through a 3 to 5 cm incision between the anterior and mid-axillary lines at the fourth or fifth intercostal space; placement of the chest drainage tube on the dorsal side of the incision; and the chest tube size was 28 F.

Exclusion criteria included: patients with hypertension, coagulopathy, renal disease, chronic obstructive pulmonary disease, or those on steroid therapy; patients with a history of thoracic surgery; patients with a history of chest trauma; presence of pleural adhesions found during surgery; placement of >1 chest drainage tube; patients requiring reoperation postoperatively. The clinical data and follow-up information of the patient are incomplete.

### 2.1. Surgical procedures

All patients underwent general anesthesia with double-lumen endotracheal intubation. The incision, located between the fourth or fifth rib at the mid-axillary line, was approximately 4 cm long, extended to 5 cm for obese patients. Depending on the location of the pulmonary lesion, surgical procedures included wedge resection, segmentectomy, lobectomy, or extended resection, with the decision to perform mediastinal lymph node dissection based on intraoperative frozen section analysis. A 28 F drainage tube (manufacturer: Suzhou Tianping Huachang Medical Instruments Ltd., license number: 20020079, size: 28 F) was placed on the dorsal side of the incision, connected to a single-chamber pleural drainage bottle (manufacturer: Suzhou New District Jingxin Medical Supplies Co., Ltd., single chamber).

### 2.2. Modified chest tube drainage strategy

**Figure 1.**
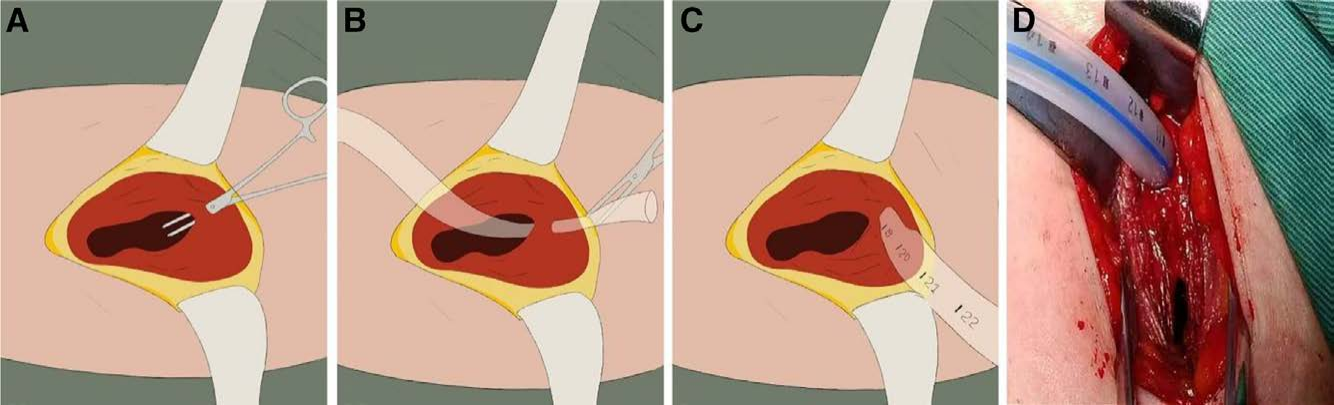
Modified chest tube drainage strategy. (A) A hemostat is used to bluntly enter the thoracic cavity from the subcutaneous tissue approximately 0.5 to 1 cm from the dorsal side of the incision after releasing the retractor, allowing the skin and subcutaneous muscle to completely cover the entry point, achieving a misalignment effect. (B, C) The distal end of the chest tube is inserted through the incision, clamped with a hemostat, and them directed out from the dorsal side of the incision. (D) Intraoperative picture after chest tube placement.

In the modified group, at the intercostal space where the uniportal access was made, a retractor is used to separate the skin and subcutaneous muscle at the posterior edge of the incision, exposing the intercostal muscle of the same rib. A hemostat is then used to bluntly enter the thoracic cavity from the subcutaneous tissue approximately 0.5 to 1 cm from the dorsal side of the incision after releasing the retractor, allowing the skin and subcutaneous muscle to completely cover the entry point, achieving a misalignment effect (Fig. 1A). The distal end of the chest tube is inserted through the incision, clamped with a hemostat, and then directed out from the dorsal side of the incision (Fig. 1B, C). The distal end of the chest tube is placed 2 cm from the pleural apex. The muscle layer is sutured, followed by interrupted sutures of the subcutaneous layer to ensure there are no dead spaces and the tension is appropriate. Then, a barbed suture (Covidien, VLOCL0315) is used to stitch the skin from the chest side to the tube, by passing it at the tube’s location, and exiting 1 to 2 cm from the dorsal side of the skin to facilitate tightening after the chest tube is removed.

### 2.3. Traditional chest tube drainage strategy

**Figure 2.**
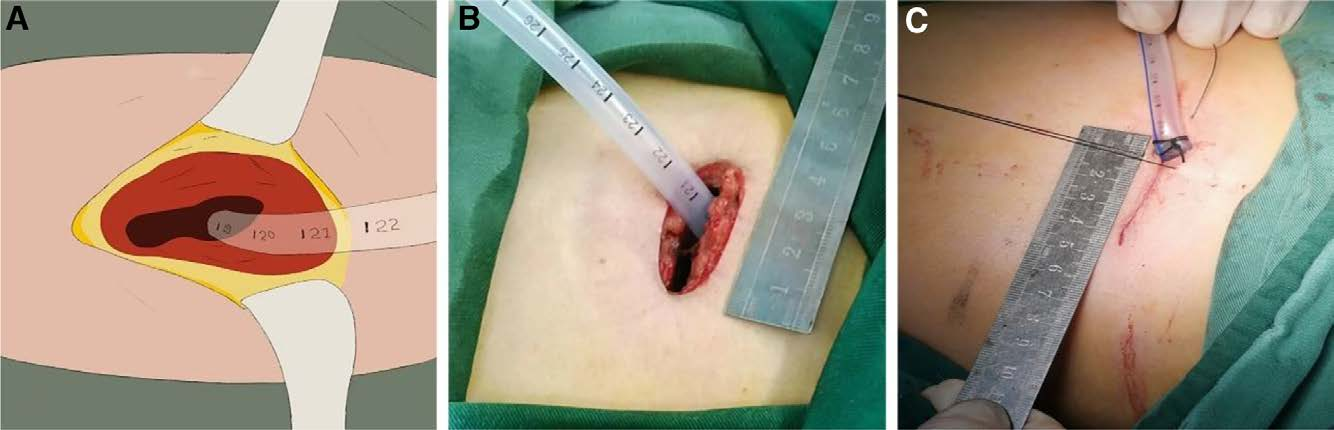
Traditional chest tube drainage strategy. (A) The chest tube is directly exited from the dorsal side of the incision. (B) Intraoperative picture after chest tube placement. (C) A suture is placed in reserve at the site of the tube, which is tied off after the chest tube is removed.

In the control group, the chest tube is directly exited from the dorsal side of the incision (Fig. 2A, B), with the muscle, subcutaneous tissue, and skin all sutured in the same manner. A suture is placed in reserve at the site of the tube, which is tied off after the chest tube is removed, or the skin is re-sutured at the time of tube removal (Fig. 2C).

During the procedure, the incision length was measured with a straight ruler, and the width of the posterior aspect of the incision was measured with a vernier caliper. Record the surgical time.

### 2.4. Postoperative analgesia

Both groups received routine analgesia with ketorolac tromethamine (30 mg IV infusion, twice daily for 5 days), supplemented by rescue analgesia with morphine (3–5 mg IV boluses as needed). Pain control was maintained to ensure resting (VAS-R) and dynamic (VAS-D) VAS scores remained ≤40 mm during hospitalization.^[8]^

### 2.5. Indications and method of chest tube removal

A follow-up chest x-ray shows good lung re-expansion, and there are no signs of pneumothorax or pleural effusion, no air leakage is observed in the chest drainage bottle when coughing, and the 24-hour drainage volume is <200 mL.

In both groups, patients performed the Valsalva maneuver during chest tube removal. In the end-inspiration removal group, all patients practiced the procedure at least once before actual removal. In the traditional group, the incision was closed by tying the pre-placed suture and securing the barbed suture after tube removal, whereas the modified group involved direct tube removal with barbed suture tightening only. After tube removal, all patients had the wound site covered with Vaseline gauze and sterile gauze, secured with cloth tape. Within 2 to 4 hours after removal, all patients underwent posteroanterior chest radiography in the radiology department.^[9]^

### 2.6. Follow-up and evaluation criteria

Postoperative pain scores were observed at rest and during activity on days 1, 2, and 3, as well as at rest after tube removal. Observations included the postoperative time of operation, drainage duration, volume of drainage, extubation operation time, complications of extubation, morphine recovery times, postoperative hospital stay and drainage tube site sound healing.

On the day of surgery, patients routinely used a patient-controlled analgesia pump. Pain was assessed daily using the visual analog scale (VAS), with a vertical numeric rating ranging from 0 to 10, where 0 indicates no pain; 1 to 2 indicates occasional mild pain; 3 to 4 denotes frequent mild pain; 5 to 6 signifies occasional severe pain that is tolerable; 7 to 8 represents frequent severe pain that is still tolerable; and 9 to 10 denotes excruciating, intolerable pain. Scores under stable conditions were considered rest pain ratings, while pain scores post chest tube stimulation, coughing, or physical activity were considered activity pain ratings. Pain conditions within the first 72 hours postoperatively were recorded.^[10]^

Drainage tube site wound healing was categorized into A, B, or C levels. Grade A healing was good with no adverse reactions; grade B healing involved poor healing with inflammatory reactions including redness, hardening, hematoma, and effusion, but no suppuration; grade C healing indicated suppurative wounds requiring debridement and drainage. Sutures were generally removed 10 to 14 days after chest tube removal, depending on the wound healing status.

Complications of chest tube removal include: wound exudate, pneumothorax, subcutaneous emphysema, and reinsertion of the chest tube.^[11]^

Extubation operation time is defined as the period starting from the beginning of extubation until the wound is fully dressed.

#### 2.6.1. Evaluation of CPSP

Chronic postsurgical pain (CPSP), as classified by ICD-11, refers to persistent pain that either emerges or worsens following surgical intervention or tissue damage, continuing for at least 3 months after the initial trauma or procedure. This condition extends beyond the expected recovery period and differs from preoperative discomfort.^[12]^

To assess CPSP occurrence post-VATS, telephone interviews were conducted using a structured questionnaire. The evaluation included the following key aspects^[13]^:

1. Presence of persistent pain near the surgical site or adjacent areas 3 months postoperatively.
2. Comparison between postoperative pain and any preexisting preoperative discomfort.
3. Exclusion of alternative pain etiologies (such as tumor recurrence or chronic infections).
4. Pain intensity quantification using an 11-point numerical rating scale (0 = no pain, 10 = maximum imaginable pain).
5. Pain management strategies employed (including activity modification, self-medication, or medical consultation).

Patients reporting numerical rating scale scores ≥1 for their worst pain experience during rest, movement, or other activities were considered positive for CPSP diagnosis. This threshold was implemented to capture all potential cases, including those with mild symptoms.^[14]^

#### 2.6.2. Scar assessment

The patient and observer scar assessment scale (POSAS) were used for the assessment of scars by a specially trained physician at the outpatient clinic 1 year after surgery. The POSAS consists of 2 distinct evaluation tools^[15]^:

1. Observer scar assessment scale – assessed by clinicians, this scale examines 5 scar attributes: vascularization, thickness, relief, pigmentation, and pliability. Scores range from 5 (minimal scarring) to 50 (most severe scarring).
2. Patient scar assessment scale (PSAS) – completed by patients, this scale evaluates 6 subjective scar characteristics: pain, itching, color, stiffness, thickness, and irregularity. The scoring system spans from 6 (normal skin) to 60 (worst possible scar).

This dual approach ensures comprehensive scar evaluation from both clinical and patient-reported perspectives.

A 1-year postoperative follow-up including chest computed tomography and pulmonary function tests revealed encapsulated pleural effusion, with concurrent assessment of respiratory function.

### 2.7. Statistical analysis

In this study, the matching variables were selected based on their potential influence on postoperative pain and recovery outcomes, including demographic factors (age, gender), clinical parameters (BMI, smoking status, pulmonary function), and surgical characteristics (resection extent, incision width, pathology). These covariates were included to minimize confounding bias. The caliper value was set at 0.2 standard deviations of the logit of the propensity score, consistent with established methodological recommendations, to balance bias reduction with retention of sample size.

Continuous data adhering to normal distribution and homogeneity of variance were compared between the 2 groups using the independent samples *t* test, represented by mean ± standard deviation; non-normally distributed data were compared using the Mann–Whitney *U* test, represented by median and quartile ranges; categorical data were analyzed using the Chi-square test or Fisher’s exact test, represented by frequency (rate). Bilateral tests were conducted, with *P* < .05 considered statistically significant. All analyses were performed using R software version 4.5.0.

## 3. Results

### 3.1. PSM results

After propensity score matching on 160 patients in traditional group and the 46 patients in modified group, 40 patients in each group were finally matched (“caliper = 0.2”). Before matching, the baseline characteristics of 2 groups were unbalanced, with statistically significant differences in gender (*P* = .029), Fev1 (*P* = .016), width of the incision (*P* = .017) and pathology (*P* = .042). While after matching, the differences in the fourteen confounding factors were not statistically significant between the 2 group (all > 0.05), and the baseline characteristics of patients were balanced, as shown in Table 1.

**Table 1:** Comparison of baseline information before and after propensity score matching between the 2 group (case/*x̄* ± *s*).

Characteristics	Before PSM			After PSM		
	Traditional group (n = 160)	Modified group (n = 46)	*P*	Traditional group (n = 40)	Modified group (n = 40)	*P*
Gender			.029			.361
Male	105	22		26	22	
Female	55	24		14	18	
Age (yr)	59.56 ± 12.34	61.28 ± 9.84	.384	59.98 ± 11.90	60.68 ± 9.93	.682
BMI (kg/m^2^)	20.57 ± 1.48	20.97 ± 1.63	.109	20.88 ± 1.42	20.94 ± 1.74	.861
Smoking			.469			.201
Yes	15	6		1	5	
No	145	40		39	35	
Drinking			.541			.239
Yes	23	5		9	5	
No	137	41		31	35	
Diabetes			.081			.737
Yes	33	4		6	4	
No	127	42		34	36	
ALB	40.77 ± 4.41	41.11 ± 3.84	.640	40.96 ± 3.89	41.51 ± 3.89	.529
FVC (L)	3.30 ± 0.30	3.25 ± 0.28	.345	3.29 ± 0.28	3.26 ± 0.28	.612
FEV1	2.66 ± 0.16	2.59 ± 0.17	.016	2.64 ± 0.18	2.60 ± 0.18	.376
FEV1%	84.75 ± 2.59	84.71 ± 2.88	.940	85.68 ± 2.62	84.96 ± 2.99	.251
Width of the incision	2.01 ± 0.17	1.93 ± 0.21	.017	2.03 ± 0.22	1.97 ± 0.20	.210
Length of the incision	3.91 ± 0.20	3.90 ± 0.23	.901	3.91 ± 0.20	3.90 ± 0.24	.891
Extent of resection			.655			.428
Wedge resection	36	8		6	8	
Segmentectomy	9	1		2	1	
Lobectomy	105	33		32	28	
Extended resection	10	4		0	3	
Pathology			.042			.412
Benign	27	14		7	10	
Malignancy	133	32		33	30	
Lymphadenectomy			.326			.446
Yes	106	34		28	31	
No	54	12		12	9	

ALB = albumin, BMI = body mass index, FEV1 = forced expiratory volume in 1 second, FVC = forced vital capacity, PSM = propensity score matching.

The surgical data of the 2 group of patients after propensity score matching are specified in Table 2. Both groups of surgeries were completed successfully. Significant differences were observed in postoperative pain, extubation operation time, complications of extubation, morphine rescue times and drainage tube site wound healing (*P* < .05), with the modified group showing superior outcomes compared to the traditional group.

**Table 2:** Surgical data of patients (case/*x̄* ± *s*).

Characteristics	Traditional group (n = 40)	Modified group (n = 40)	*P*
Time of operation (min)	128.40 ± 36.90	133.38 ± 41.42	.572
Rest VAS score (POD1)	4.44 ± 1.34	3.82 ± 1.27	.036
Rest VAS score (POD2)	3.43 ± 0.98	2.70 ± 0.99	.001
Rest VAS score (POD3)	2.39 ± 0.65	1.54 ± 0.52	<.001
Active VAS score (POD1)	5.77 ± 0.80	4.99 ± 0.84	<.001
Active VAS score (POD2)	4.81 ± 0.53	3.73 ± 0.66	<.001
Active VAS score (POD3)	3.65 ± 0.43	2.75 ± 0.44	<.001
Extubation VAS score	4.90 ± 0.77	2.47 ± 0.90	<.001
drainage duration (d)	5.35 ± 1.81	5.30 ± 1.84	.763
Volume of drainage (mL)	576.93 ± 253.27	589.58 ± 265.78	.828
Extubation operation time (s)	68.55 ± 5.60	30.95 ± 4.01	<.001
Complications of extubation			.018
Yes	14	5	
No	26	35	
Morphine rescue times	2 (1, 2.25)	1 (0, 2)	.016
Postoperative hospital stay (d)	6.40 ± 1.72	6.35 ± 1.76	.898
Drainage tube site wound healing			.032
Grade A	27	36	
Grade B	11	4	
Grade C	2	0	

POD = postoperative day, VAS = visual analog scale.

One year after surgery, follow-up evaluations revealed no statistically significant differences between the 2 groups in the incidence of encapsulated pleural effusion or lung function parameters (forced vital capacity, forced expiratory volume in 1 second, forced expiratory volume in 1 second %). However, the modified surgical technique group demonstrated significantly lower chronic incision pain and better scar healing scores compared to the traditional group (*P* < .05; Table 3).

**Table 3:** One-year postoperative follow-up outcomes after propensity score matching (case/*x̄* **±** *s*).

Characteristics	Traditional group (n = 40)	Modified group (n = 40)	*P*
Encapsulated pleural effusion			.615
Yes	1	3	
No	39	37	
CPSP			.029
Yes	8	1	
No	32	39	
Scar assessment			
OSAS	12.50 ± 1.74	10.75 ± 2.19	<.001
PSAS	11.55 ± 1.55	8.95 ± 1.69	<.001
Postoperative FVC	3.00 ± 0.25	2.98 ± 0.28	.702
Postoperative FEV1	2.30 ± 0.17	2.28 ± 0.18	.607
Postoperative FEV1%	83.26 ± 2.69	82.68 ± 3.02	.369

CPSP = chronic postsurgical pain, FEV1 = forced expiratory volume in 1 s, FVC = forced vital capacity, OSAS = observer scar assessment scale, PSAS = patient scar assessment scale.

## 4. Discussion

In 2004, Rocco^[16]^ first applied uniportal video-assisted thoracoscopic surgery (U-VATS) to lung wedge resections. Due to its minimal invasiveness, aesthetically pleasing incisions, and reduced postoperative pain, it has become increasingly accepted by surgeons and patients alike. Uniportal VATS has evolved from initially addressing pneumothorax, pleural diseases, and lung biopsies to now being capable of performing radical lung cancer surgeries, mediastinal tumor resections, and even complex procedures such as sleeve resections and tracheobronchial reconstructions.^[17,18]^

Transitioning from traditional open thoracotomy to U-VATS, the routine placement of a chest drainage tube remains the preferred method by most surgeons to drain pleural effusions and air. The chest tube is a primary source of postoperative pain and a major cause of patient dissatisfaction with cardiothoracic surgery.^[19]^ A single 28 F chest tube, known for its high and consistent flow rate, maintains a good balance between size and flow efficiency and has been proven to be an effective drainage method.^[20]^ Typically made of silicone, these tubes are relatively rigid, thick, and inflexible. They are inserted through narrow intercostal spaces, compressing the intercostal nerves, causing pleural irritation due to friction between the tube and surrounding tissues during movement or breathing. Additionally, to prevent leakage around the tube, the surrounding wall is often sutured tightly, creating significant tissue tension. These factors are significant contributors to postoperative pain and poor incision healing. Pain induced by the chest tube can hinder patient mobility, including deep breathing, coughing, and early ambulation, thereby affecting rapid recovery.^[21]^ Various factors such as suture techniques, patient nutrition, and duration of chest tube placement can differently impact wound healing.

To date, there is no recommended method for incision closure or chest tube placement in U-VATS. Minimizing postoperative pain and complications related to poor wound healing without compromising drainage efficiency remains a significant area of research. This study aims to explore an improved technique for chest tube insertion to enhance its clinical application.

The improved method of chest tube placement involves bluntly dissecting the intercostal muscles to establish a new pathway without severing the muscles, thus preserving the integrity of the intercostal tissues. The intercostal muscles at the surgical incision are then interruptedly sutured to close the incision. This modification reduces friction between the chest tube and the tissues at the incision site, and diminishes irritation to the pleura. Traditionally, chest tubes are inserted directly through the incision to maintain airtightness and prevent leakage of fluids or air around the incision, often requiring densely placed sutures with significant tension.^[22]^ To close the incision after tube removal, additional reserve sutures are needed, which when tightened can cause pain due to their cutting effect on the tissue. With the improved technique, the intercostal muscles naturally retract after the chest tube is removed, closing the pathway between the thorax and the external environment naturally, eliminating the need for sutures and effectively reducing pain, leakage, and other complications associated with tube removal.

Furthermore, we employ a method of continuously suturing the skin using absorbable, knotless barbed sutures. Barbed suture material, now widely used across various surgical fields, has proven effective without increasing morbidity.^[23,24]^ V-LOC, a suture composed of a copolymer of glycolide and trimethylene carbonate, retains about 80% of its tensile strength at 2 weeks and approximately 30% at 3 weeks, with complete absorption occurring between 56 and 70 days. This suturing technique has achieved satisfactory results.^[25–27]^ After the chest tube is removed, simply pulling the barbed suture on one side sufficiently tightens the skin. The use of absorbable, knotless barbed sutures reduces the amount of foreign material in the wound, shortens closure time, enhances the cosmetic appearance of the incision, and does not compromise the strength of the incision.^[28]^

Our experience is as follows: we recommend using interrupted sutures for the subcutaneous and muscle layers on both sides of the wound to prevent leakage near the drainage tube; hemostats should enter the thoracic cavity bluntly through the middle of the intercostal space to avoid damaging the intercostal vessels and nerves; the entry point of the hemostat from the outside to the inside of the thoracic cavity should be approximately 0.5 to 1 cm from the dorsal side of the incision. This distance should be adjusted based on the patient’s skin tension and elasticity. Too great a distance can increase the tension around the skin of the drainage tube, potentially causing the tube to bend.

The 8-fold reduction in CPSP incidence with our modified technique (2.5% vs 20%, *P* = .029) integrates multiple evidence-based mechanisms. First, the significantly attenuated *activity-related pain* (VAS-D POD 1–3, *P* < .001) is clinically critical, as Wang et al^[29]^ demonstrated dynamic pain during coughing/movement predicts CPSP persistence more strongly than rest pain. Second, by avoiding direct intercostal nerve compression through the “misalignment” design (Fig. 1A), we address Zhang et al’s^[13]^ identification of *drainage-related trauma* as a key CPSP risk factor (OR = 2.48). This aligns with Chen et al’s^[30]^ meta-analysis confirming that minimizing surgical insult reduces CPSP prevalence (pooled OR = 0.41, 95% CI: 0.29–0.58). Crucially, our acute pain control strategy – lowering VAS-R/VAS-D scores – directly interrupts the *acute-to-chronic transition* pathway emphasized by Tong et al,^[12]^ where early VAS > 4 increases CPSP risk by 3.2-fold. Collectively, these refinements operationalize Bendixen et al’s^[14]^ enhanced recovery after surgery (ERAS) principles, extending VATS’ minimally invasive advantages from immediate recovery to long-term pain-related quality of life. Chronic pain transition. Collectively, these refinements extend ERAS principles beyond the immediate perioperative period, offering long-term quality-of-life benefits.

Our modified strategy demonstrated significantly better scar outcomes at one year follow-up, evidenced by lower POSAS scores (observer scar assessment scale: 10.75 ± 2.19 vs 12.50 ± 1.74; PSAS: 8.95 ± 1.69 vs 11.55 ± 1.55, *P* < .001). This improvement stems from 2 synergistic innovations: First, the barbed suture technique minimizes tissue trauma and suture tension – validated by Kim^[7]^ who reported reduced inflammation and improved cosmesis in uniportal VATS closures using similar methods. Second, the separate drainage pathway (Fig. 1A) eliminates direct tube-skin friction, preventing focal pressure necrosis observed in traditional exits (Fig. 2B). Crucially, the PSAS reduction (>2.5-point difference) exceeds the minimal clinically important difference (MCID = 1.5), indicating patients perceived substantially less scar stiffness and itching – a critical quality-of-life factor emphasized by Fu et al.^[15]^ These findings align with ERAS principles by transforming drainage management from a functional necessity to an aesthetic optimization, particularly valuable for young patients or those with keloid predisposition. Beyond the statistical outcomes, our findings can be explained by potential mechanisms. The misalignment design in the modified drainage strategy reduces direct compression of intercostal nerves, minimizing local tissue trauma and inflammatory responses. These effects may contribute to reduced acute pain, lower incidence of CPSP, and improved wound healing. Additionally, by reducing tissue irritation and promoting better scar outcomes, the modified strategy has the potential to improve long-term quality of life. When compared with international studies, our results are consistent with findings that modified drainage techniques can alleviate postoperative pain, but our inclusion of 1-year CPSP and scar outcomes offers novel insights. Nevertheless, our single-center retrospective design and relatively small matched sample size limit the external validity of the conclusions. Larger multicenter trials are warranted.

Given that this is a preliminary retrospective study, it has significant limitations, including its retrospective nature and potential for subjective bias, variations caused by different team members, lack of documentation on the duration of each suturing, insufficient follow-up time, and the necessity for additional scar assessments.

## 5. Conclusion

The modified chest drainage tube suturing technique and tube fixation method described in this paper are safe, effective, and cosmetically appealing. Moreover, As an essential element in the ERAS program, this technique significantly contributes to postoperative recovery in thoracic surgery cases. We believe that this improved tube suturing and fixation technique will be increasingly applied in more patients.

## Author contributions

**Conceptualization:** Min Hu, Jing Li, Baochen Zhao, Haoran Sun.

**Data curation:** Haoran Sun.

**Formal analysis:** Min Hu, Baochen Zhao.

**Investigation:** Min Hu, Jing Li, Baochen Zhao, Haoran Sun.

**Methodology:** Min Hu, Jing Li, Baochen Zhao, Haoran Sun.

**Supervision:** Jing Li, Baochen Zhao, Haoran Sun.

**Validation:** Min Hu, Jing Li, Baochen Zhao, Haoran Sun.

**Visualization:** Min Hu, Jing Li, Baochen Zhao, Haoran Sun.

**Writing – original draft:** Min Hu, Jing Li.

**Writing – review & editing:** Min Hu, Jing Li.
